# Acupuncture for perimenopausal depressive disorder: A systematic review and meta-analysis protocol

**DOI:** 10.1097/MD.0000000000014574

**Published:** 2019-02-15

**Authors:** Jialei Feng, Wei Wang, Yuan Zhong, Chonghui Xing, Taipin Guo

**Affiliations:** a Yunnan University of Chinese Medicine, Kunming, Yunnan; b Hospital of Traditional Chinese Medicine Affiliated to the Southwest Medical University, Luzhou, Sichuan; c The Sports Trauma Specialist Hospital of Yunnan Province, Kunming, China.

**Keywords:** acupuncture, meta-analysis, perimenopausal depressive disorder, protocol, systematic review

## Abstract

**Background:**

Perimenopausal depressive disorder (PDD) is a disease that plagues many perimenopausal women. There is an urgent need for a safe way to treat the disease. With few side effects, acupuncture treatment for PDD has been gradually accepted. However, at present, the evidence is insufficient and relevant studies are not in-depth enough. The purpose of this study is to explore the efficacy and safety of acupuncture for PDD.

**Methods:**

All randomized controlled trials articles on acupuncture treatment of PDD will be searched in databases such as MEDLINE, EBASE, Cochrane Library, Springer, World Health Organization International Clinical Trials Registry Platform, China National Knowledge Infrastructure, Wan Fang, Chinese Biomedical Literature Database, Chinese Scientific Journal Database and so on. Non-RCT articles will be screened and key information will be extracted. The primary outcome is the Hamilton depression scale. Second outcomes are the Hamilton anxiety scale, Quality of life scale, changes of symptoms in traditional Chinese medicine and hormone levels.

**Results:**

This systematic review will provide the highest level of evidence and provide an evaluation of the efficacy and safety of acupuncture for PDD.

**Conclusion:**

This study provides evidence for evaluating the efficacy and safety of acupuncture in the treatment of PDD.

**PROSPERO registration number:**

CRD42018115811.

## 1 Introduction

### 1.1 Description of the condition

Perimenopausal depressive disorder (PDD) is a kind of mental disorder that usually occurs in women between 45 and 55 years old who are during perimenopause. Clinically, it is mainly manifested as anxiety, depression, insomnia, somatic discomfort, vegetative nervous dysfunction, and other symptoms, as well as changes of endocrine function. Patients without a clear diagnosis may also experience jealousy, delusional, and self-abased.^[1–3]^ The duration of PDD is 2 to 3 years, and the length can be up to more than 10 years, which seriously endangers the physical and mental health of women in this period, affects the daily work and life of patients, and also causes a burden on families and society.^[4,5]^ By 2012, there are 350 million people worldwide were suffering from depression, about 6.7% of them were in the United States.^[6]^ With the increasing pressure of life, depressive disorder is expected to become the second of the world's top 10 diseases and the second leading cause of disability in the world by 2020. The incidence of depressive disorder in women is 1.5 to 3 times higher than men and is increasing year by year.^[3,7,8]^ In the world, the incidence of a menopausal syndrome is 28.5%.^[5]^ In China, perimenopausal women account for about 10% of the total population and will continue to rise.^[9]^ The incidence of PDD in perimenopausal women can reach 9.24%.^[4]^ Currently, the prevalence rate of this disease among Chinese women is increasingly higher, about 21%.^[10]^

In order to improve the quality of life of perimenopausal women, a safe, convenient, and lasting treatment is necessary, not only to treat the symptoms but also to regulate the patient's psychology.

### 1.2 Description of the intervention

Currently, traditional treatments for PDD are primarily hormones (such as nilestriol) and antidepressants (such as Prozac), but there are many side effects and risks associated with remission of clinical symptoms.^[5]^ In addition to drug treatment, this disease also emphasizes the importance of prevention, advocates active health education, advocates an optimistic attitude towards life, and timely psychological intervention.^[11]^

People are long for a safe and non-drug therapy. Traditional Chinese medicine (TCM) methods, such as acupuncture, tuina, and guasha, have been accepted by more and more people.^[12]^ Multiple studies have shown that acupuncture has a good therapeutic effect on depression, and can effectively regulate the mental state and behavior of patients and improve their quality of life.^[8,13–15]^

Acupuncture, which has a history of more than 3000 years in China, uses a thin silver needle to pierce a specific point in the body to treat diseases. There are 14 meridians and 361 acupoints, and the prescription of acupuncture is consisting of more than 1 acupoint which is selected based on TCM syndrome differentiation theory. Compared with other treatments, acupuncture has the advantages of safety, convenience, fewer side effects, and significant efficacy.

### 1.3 How the intervention might work

Although the mechanism of acupuncture for PDD is not clear, there is a lot of evidence that acupuncture has a good effect on depression.^[16]^ According to TCM syndrome differentiation theory, the main pathogenesis of PDD are kidney deficiency with liver-qi stagnation and heart-kidney imbalance with liver depression, which are closely related to heart, liver, kidney, and spleen.^[17]^ Acupoints are often used to treat emotional diseases because of their functions of nourishing the kidney, nourishing the heart, soothing the liver, relieving depression, and calming the mind. Besides, the study has shown that acupuncture affects estrogen receptors, affects their expression and regulates estrogen levels in women.^[18]^

### 1.4 Why it is important to perform this review?

Although there is evidence that acupuncture is effective for depression,^[16]^ studies on acupuncture treatment of PDD have not gone far enough. A systematic review provides the highest level of evidence and provides an evaluation of the efficacy and safety of 1 treatment for a disease. So, it is important to perform this study.

### 1.5 Objectives

The purpose of this systematic study is to evaluate the efficacy and safety of acupuncture for PDD, which may provide the evidence and form a treatment recommendation for clinicians and researchers.

## 2 Methods

### 2.1 Study registration

PROSPERO systematical review protocol registration number is CRD42018115811. This protocol will comply with the preferred reporting items for systematic reviews and meta-analyses protocols (PRISMA-P) statement guidelines.^[19]^

### 2.2 Inclusion criteria for study selection

#### 2.2.1 Types of study

To assess the efficacy and safety of acupuncture for PDD, all related RCTs will be included in the evaluation. This review includes only randomized controlled trials (RCTs) of acupuncture on PDD, which will be categorized by language in Chinese and English. Others such as case reports, animal experiments, non-RCTs, or RCT protocol will be excluded.

#### 2.2.2 Types of participants

The patient range is limited to perimenopausal women, the cause of PDD is not limited. The diagnostic criteria can be included with or without perimenopausal symptoms.

#### 2.2.3 Types of intervention

This study focuses on clinical trials of acupuncture on PDD, and the results will be recommended to clinicians. Therefore, different types of acupuncture operations will be included, including body acupuncture, ear acupuncture, electroacupuncture, acupoint embedding, and so on. The comparisons between acupuncture and other treatments and the comparisons between different methods of acupuncture will also be included. Combinations of treatments that cannot evaluate the efficacy of acupuncture will be excluded.

#### 2.2.4 Types of outcome measures

The primary outcome is the Hamilton depression scale (HAMD). Second outcomes are the Hamilton anxiety scale (HAMA), quality of life scale (SF-36), changes of symptoms in TCM, and hormone levels.

### 2.3 Data sources

English databases such as MEDLINE, EBASE, Cochrane Library, Springer, World Health Organization International Clinical Trials Registry Platform, and Chinese databases such as China National Knowledge Infrastructure, Wanfang, Chinese Biomedical Literature Database, and Chinese Scientific Journal Database will be searched according to their own search rules.

### 2.4 Search strategy

The acupuncture terms or keyword combinations acupuncture and similar words (eg,“acupuncture” or “electro-acupuncture” or “ear acupuncture”, etc) will be combined with the disease terms or keyword combinations (eg, “menopause depression” or “climacteric depression” or “perimenopausal syndrome” or “involutional depression, etc”). Medline's search strategy is shown in Table 1.

**Table 1:**
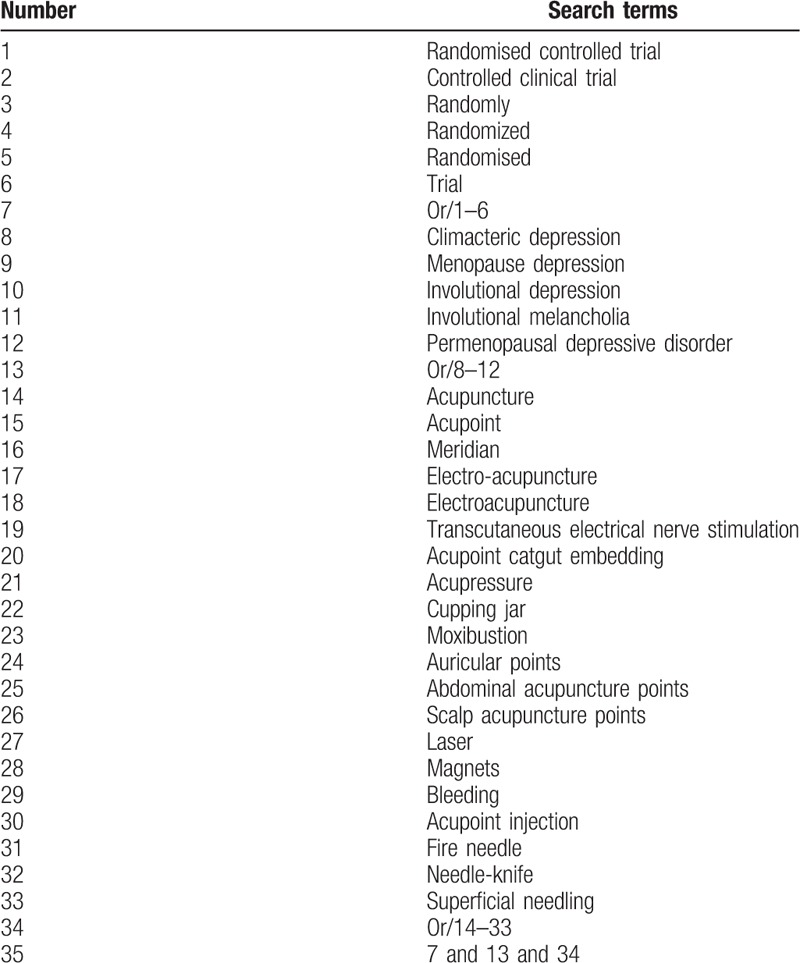
Medline search strategy.

### 2.5 Data collection and analysis

#### 2.5.1 Selection of studies

**Figure 1:**
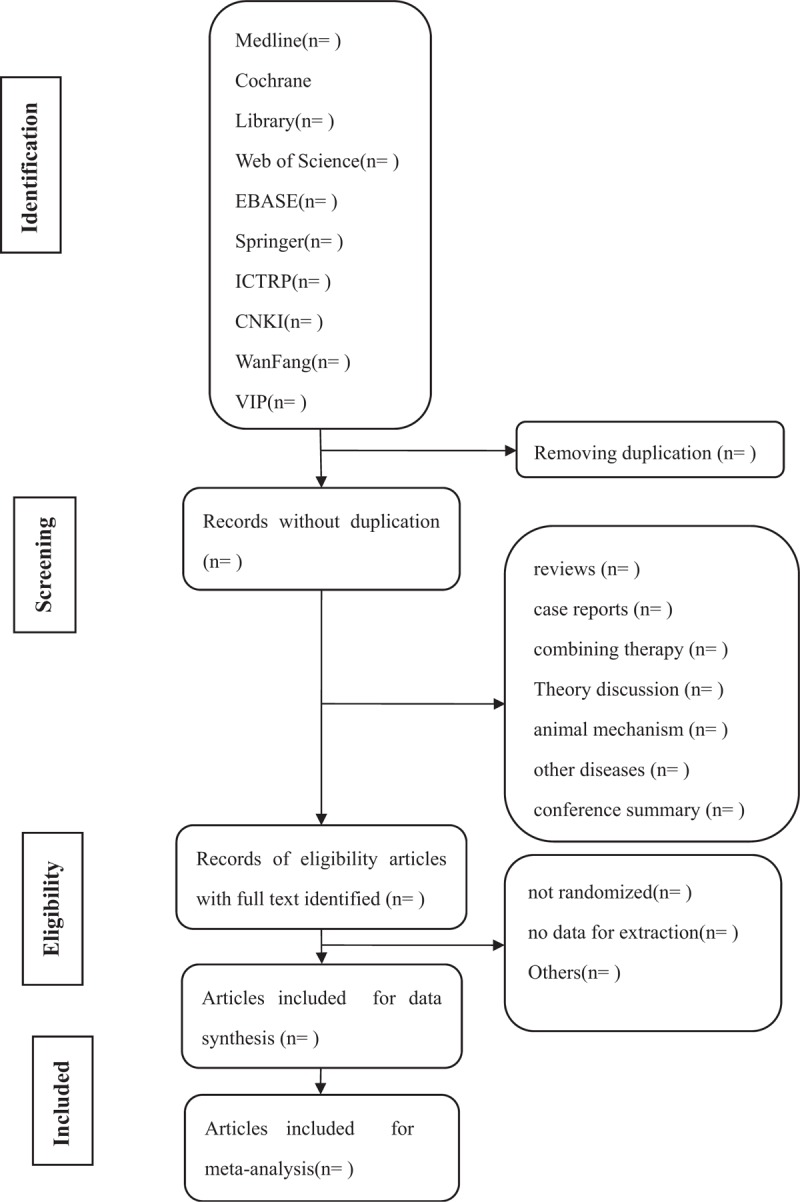
Flow diagram of studies identified.

The 2 researchers will conduct data collection, data analysis, and quality evaluation independently as 2 parts. The full text of all relevant articles will be filtered. A third researcher will make the final decision when there is disagreement between the 2 researchers and difficulty in reaching an agreement. The main selection process is shown in the PRISMA flow chart (Fig. 1).

#### 2.5.2 Data extraction and management

Data on study design, patient characteristics, acupuncture intervention, and control intervention will be extracted by 2 researchers and recorded in electronic documents, and all information will be re-examined crossly. The third researcher will have a divergent discussion.

#### 2.5.3 Assessment of risk of bias

We will use the Cochrane bias risk tool (RevMan V.5.3; Copenhagen, The Nordic Cochrane Centre, The Cochrane Collaboration, 2014)^[20]^ to evaluate the bias risk.

#### 2.5.4 Measures of treatment effect

Mean differences of 95% confidence interval (95%CIs) will be used as continuous data, and risk ratio will also be used as a dichotomous data expression.

#### 2.5.5 Unit of analysis issues

Based on the results, the HAMD scales will be aggregated and secondary results will be analyzed separately, including the HAMA scale, SF-36, TCM symptom changes, and hormone levels.

#### 2.5.6 Management of missing data

If there is missing or incomplete data, we will first try to contact the original author. If the data cannot be provided, then the malformed data will be get rid of.

#### 2.5.7 Assessment of heterogeneity

This study will use the chi-squared test to calculate the heterogeneity, and the degree of heterogeneity depends on the value of *I*^2^. The results showed that when *I*^2^ > 50% it will show substantial heterogeneity, and the random effect model will be chosen. If *I*^2^ ≤ 50%, it states no heterogeneity, and the fixed-effect model will be chosen.

#### 2.5.8 Assessment of reporting biases

When more than 10 trials are included, the funnel plot will be used to evaluate the reported biases. Its symmetry can explain the biases. If the funnel plot is symmetric, it indicates that there are no reported biases; otherwise, it means there are biases.

#### 2.5.9 Data synthesis

RevMan version 5.3 will be used to analyze 95%CI as a quantitative analysis. The average change in each primary and secondary result will be merged. If the data is not suitable for quantitative analysis, qualitative analysis will be used.

#### 2.5.10 Subgroup analysis

Subgroup analysis will be handled according to the differences in acupuncture methods, patient conditions, and control.

#### 2.5.11 Sensitivity analysis

Our sensitivity analysis will be based on heterogeneity and predefined criteria.

## 3 Discussion

PDD increases women's mental stress and can seriously affect their quality of life. In addition, the increasing incidence of PDD will also place a burden on families and society. Although the mechanism of acupuncture as a non-drug therapy for the treatment of PDD is not yet clear, its curative effect has been proved, with few adverse reactions and almost no side effects.^[16]^ The lack of relevant evidence has led to limited promotion worldwide. It is still necessary to conduct the study, although some potentially low-quality raw RCT may affect the study's accuracy. The study, which will bring together all Chinese and English RCT studies involving different acupuncture methods to treat perimenopausal depression, will provide evidence of efficacy and safety and promote the use of acupuncture for perimenopausal depression.

## Author contributions

**Conceptualization:** Wei Wang.

**Data curation:** Jialei Feng, Yuan Zhong, Chonghui Xing, Tainpin Guo.

**Formal analysis:** Wei Wang.

**Investigation:** Tainpin Guo.

**Methodology:** Yuan Zhong, Tainpin Guo.

**Resources:** Jialei Feng.

**Software:** Wei Wang, Tainpin Guo.

**Supervision:** Chonghui Xing, Tainpin Guo.

**Validation:** Yuan Zhong.

**Visualization:** Tainpin Guo.

**Writing – original draft:** Jialei Feng, Wei Wang.

**Writing – review and editing:** Tainpin Guo.
